# School-based cognitive behavioral interventions for anxious youth: study protocol for a randomized controlled trial

**DOI:** 10.1186/s13063-017-1831-9

**Published:** 2017-03-04

**Authors:** Bente Storm Mowatt Haugland, Solfrid Raknes, Aashild Tellefsen Haaland, Gro Janne Wergeland, Jon Fauskanger Bjaastad, Valborg Baste, Joe Himle, Ron Rapee, Asle Hoffart

**Affiliations:** 1 Regional Centre for Child and Youth Mental Health and Child Welfare, Uni Research Health, Pb 7810, 5020 Bergen, Norway; 2 Clinic of Mental Health, Psychiatry and Addiction Treatment, Sorlandet Hospital HF, Kristiansand, Norway; 3 Department of Child and Adolescent Psychiatry, Division of Psychiatry, Haukeland University Hospital, Bergen, Norway; 4 Division of Psychiatry, Stavanger University Hospital, Stavanger, Norway; 5 Department of Psychiatry, University of Michigan, Ann Arbor, USA; 6 Centre for Emotional Health, Macquarie University, Sydney, Australia; 7 Research Institute, Modum Bad Psychiatric Centre, Modum, Norway; 8 Department of Psychology, University of Oslo, Oslo, Norway

**Keywords:** anxiety, adolescents, school-based, low-intensity CBT

## Abstract

**Background:**

Anxiety disorders are prevalent among adolescents and may have long-lasting negative consequences for the individual, the family and society. Cognitive behavioral therapy (CBT) is an effective treatment. However, many anxious youth do not seek treatment. Low-intensity CBT in schools may improve access to evidence-based services. We aim to investigate the efficacy of two CBT youth anxiety programs with different intensities (i.e., number and length of sessions), both group-based and administered as early interventions in a school setting. The objectives of the study are to examine the effects of school-based interventions for youth anxiety and to determine whether a less intensive intervention is non-inferior to a more intensive intervention.

**Methods/design:**

The present study is a randomized controlled trial comparing two CBT interventions to a waitlist control group. A total of 18 schools participate and we aim to recruit 323 adolescents (12-16 years). Youth who score above a cutoff on an anxiety symptom scale will be included in the study. School nurses recruit participants and deliver the interventions, with mental health workers as co-therapists and/or supervisors. Primary outcomes are level of anxiety symptoms and anxiety-related functional impairments. Secondary outcomes are level of depressive symptoms, quality of life and general psychosocial functioning. Non-inferiority between the two active interventions will be declared if a difference of 1.4 or less is found on the anxiety symptom measure post-intervention and a difference of 0.8 on the interference scale. Effects will be analyzed by mixed effect models, applying an intention to treat procedure.

**Discussion:**

The present study extends previous research by comparing two programs with different intensity. A brief intervention, if effective, could more easily be subject to large-scale implementation in school health services.

**Trial registration:**

ClinicalTrials.gov, NCT02279251. Registered on 15 October 2014. Retrospectively registered.

**Electronic supplementary material:**

The online version of this article (doi:10.1186/s13063-017-1831-9) contains supplementary material, which is available to authorized users.

## Background

Anxiety disorders are among the most prevalent mental health problems in adolescents [1, 2], often having a chronic course, and may represent a considerable burden to individuals, families and society [3–6]. Youth anxiety is associated with a decreased level of functioning in many areas, e.g., poorer academic performance, social dysfunction, sleep problems, school absenteeism and school drop-out [7–11]. Youth anxiety furthermore increases the risk for subsequent depression and substance abuse [12–14]. Thus, early identification and intervention to prevent the onset of youth anxiety disorders is critical to reduce the adverse effects of anxiety on development, social functioning and school performance.

Cognitive behavioral therapy (CBT) is an effective treatment for anxiety disorders in children and adolescents [15–17]. However, the majority of anxious adolescents do not receive treatment [18, 19], and among those who do, long delays from disorder onset to treatment are common [20]. Children and adolescents often experience considerable barriers accessing mental health services (e.g., lack of knowledge of mental health problems among care givers and health workers, referral procedures, long distances and lack of transportation, stigma, costs) [21, 22]. It is therefore of critical importance to develop, implement and evaluate easily accessible, early interventions for anxious youth.

### Low-intensity CBT

Attempts to increase access to evidence-based interventions have resulted in a shift in treatment delivery, away from face-to-face high intensity treatments by specialist mental health care professionals, toward low-intensity CBT methods (LI-CBT). No common definition of LI-CBT is found. However, Bennett-Levy et al. [23] argue that LI- CBT aims to achieve similar outcomes with *less costly and easier to access interventions* compared to standard CBT. LI-CBT typically includes briefer and/or fewer sessions, may include use of self-help material (e.g., books, internet programs), use of group interventions and treatments delivered by less specialized health care workers [23]. This approach aims to make evidence-based treatments more accessible for larger groups suffering from the most prevalent mental health problems, such as mild to moderate levels of anxiety. Despite the increased availability of LI-CBT interventions, few rigorous studies are available that have evaluated the effectiveness of these with young people.

### School-based interventions

To achieve broader dissemination of empirically validated treatments, delivering interventions to anxious youth in the school setting is an alternative to traditional treatment approaches [24]. Adolescents spend much of their time in schools, with many situations during the school day triggering anxiety (e.g., social situations, separation from primary caregivers, being evaluated by teachers and peers). School-based interventions may furthermore reduce key barriers to access help for children and adolescents.

School nurses engage with youth on a daily basis and are particularly well positioned to intervene with anxious youths. They have intimate knowledge of the school environment and represent a low-threshold health service available for all youth during school hours. However, school nurses, in line with other primary health care workers, need training to accurately identify and manage youth mental health problems [25]. Most school nurses have no prior CBT skills and limited knowledge on identifying and treating anxious youth.

Systematic reviews of studies on early intervention and targeted prevention have demonstrated that CBT is promising when delivered to children and adolescents with elevated levels of anxiety symptoms. Overall, small to moderate effects of these interventions are found [26–28]. Early intervention and targeted prevention studies most often recruit individuals with heightened levels of symptoms, but who do not necessarily fulfill the criteria for an anxiety disorder. A meta-analysis from 2011 identified 21 indicated prevention randomized controlled trials (RCTs) targeting symptoms of youth anxiety as a primary or a secondary goal [26]. The included studies varied with regard to program evaluated (primarily CBT), profession of program leaders (e.g., mental health professions, school personnel), length of intervention (usually between 8 to 12 sessions), study quality and control groups (e.g., wait-list control, attention control, no control group). The meta-analysis demonstrated that anxiety prevention programs have a significant and desirable effect for youth anxiety, with an average effect size of 0.32 for targeted prevention programs. Gender of participants was the most robust moderator for long-term effects on anxiety, with smaller effects in samples with a higher percentage of girls. The other moderators were not significant in the multivariate analysis (e.g., age of participants, profession of group leaders).

Many prevention and early intervention studies include a limited number of participants and do not provide long-term follow-up, limiting the knowledge of the long-term effectiveness of the interventions [27]. In a systematic review from 2009, focusing exclusively on school-based interventions, 11 studies of targeted prevention and early intervention were identified [28]. Only four studies included any control group (wait-list or attention control). Although the number of school-based studies has increased since this review [29, 30], we need further studies on the effect and implementation of early intervention of youth anxiety where the methodological limitations in many previous studies are addressed (i.e., insufficient sample sizes, no control groups and limited follow-up data).

The CHILLED program has previously been found to be an effective treatment for youth anxiety disorders [31, 32], with two RCTs examining the effect of the child version of the program administered as a school-based, early intervention program [29, 33]. One trial demonstrated a significant reduction in anxiety symptoms relative to a wait-list control group in children from an economically disadvantaged area (*n* = 91, 8-11 years) [33]. The other study (*n* = 152, 7-12 years) failed to find group differences on child- and teacher-reported child anxiety symptoms compared to waitlist, but found a reduction in parent-reported child anxiety symptoms [29]. No evaluation has been done examining the effect of CHILLED as early intervention in a school setting with anxious adolescents. Targeting adolescents is important, as this is a critical stage of life transition with a heightened risk of subclinical levels of anxiety developing into anxiety disorders [34].

Before commencing the present study, a school-based group CBT program for anxious youth (the Friends for Life program [35]) was implemented in four schools in Western Norway during 2012-2014 (Fjermestad, Wergeland, Bjaastad, Rogde & Haugland:Indicated prevention for anxious youth: A feasibility study in the school health services (in preparation)). The school nurses delivering the program reported that, due to high workload and shortage of time, it was challenging to administer weekly 90-min sessions over a period of 10 weeks. Others have argued that school-based CBT interventions have to be adapted to better fit with the school system [24] and the need to develop and evaluate school-based CBT interventions for anxious youth that reduce the burden on the school system [29]. One way of achieving this is by changing the treatment schedule toward briefer and fewer sessions. To our knowledge, no studies have focused on what intensity and amount of therapist face-to-face time that are needed for youth anxiety prevention programs to be effective.

The present study will add to previous research on school-based early intervention with anxious adolescents by comparing the effect of two CBT interventions of different intensity.

### Objectives

**Fig. 1:**
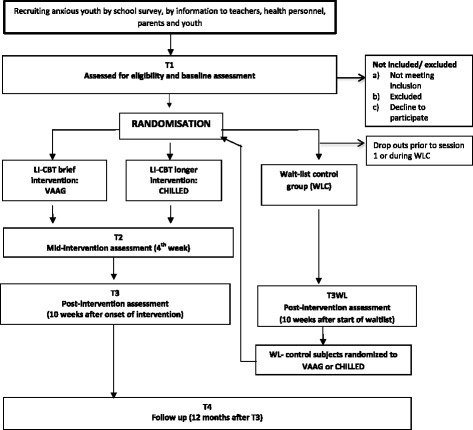
Flow chart of the study

The high prevalence and burden of anxiety for young people and the barriers to treatment for these youths make it important to evaluate low-intensity interventions that may be easy to scale up and sustain in non-specialty settings such as schools. The interventions in the present study are considered as LI-CBT because they are easily accessible, group-based and delivered primarily by school nurses. Both interventions focus on key CBT anxiety principles (e.g., psychoeducation, affect regulation, cognitive restructuring and exposure to anxiety-evoking situations). However, the two interventions differ with regard to intensity, i.e., the number and length of sessions. Research investigating the efficacy of LI-CBT for anxious youth is scarce, and it is critical to investigate whether CBT delivered by school nurses can be administered with acceptable fidelity. Moreover, we need to study with whom LI-CBT interventions should be used. In this study we aim to examine the effectiveness of two interventions for youth with anxiety (CHILLED and VAAG^1^) compared to a waitlist control group (WLC) (Fig. 1: T3, T3WL). Second, we examine whether a brief intervention (VAAG), with reduced hours of direct face-to-face therapist contact, is non-inferior to a longer intervention (CHILLED) with regard to the effect on anxiety symptoms and impairment. Finally, we investigate the long-term effects of the two CBT interventions. The main research assumptions are: (1) school-based CBT is effective for youth anxiety, (2) a brief CBT program is non-inferior to a program of higher intensity and (3) the outcome of both CBT programs will be maintained at 1-year follow-up. Our choice of a non-inferiority trial design is based on the expectation that the brief, less intensive intervention may be sufficient to make changes in adolescents with mild to moderate levels of anxiety. Also in the brief intervention a self-help component has been added that might compensate for an expected loss of effect due to lower face-to-face therapist contact (Table 1).

**Table 1:** Overview and content of the two CBT interventions

			CHILLED (school version)				VÅG
Session	participants	Duration	content	Session	participants	Duration	content
1	Adolescents	90 min	Psychoeducation. Anxiety, linking thoughts and feelings. Setting goals. Homework assignment	1	Adolescents	45 min	Psychoeducation. Anxiety, linking situations, thoughts and feelings. Setting goals Homework assignment
2^a^	Adolescents	90 min	Cognitive restructuring (“realistic thinking”) and self-rewards. Homework assignment	2	Adolescents and parents	60 min	Using self-help material to link situations, feelings and thoughts. Cognitive restructuring. Identifying avoidance. Helpful parenting. Homework assignment
3	Adolescents	90 min	Cognitive restructuring. Principles and application of exposure hierarchies (stepladders). Homework assignment	3	Adolescents	90 min	In-session exposure and behavioral experiments. Training plans. Homework assignment
4	Adolescents	90 min	Exposure hierarchy. Regulating anxiety by surfing emotions and worries. Homework assignment	4	Adolescents	90 min	In-session exposure and behavioral experiments. Training plans. Homework assignment
5^a^	Adolescents	90 min	Reviewing and revising exposure hierarchies (individual sessions 15-20 min)				
6	Adolescents	90 min	Simplifying cognitive restructuring. In-session exposure and behavioral experiments. Homework assignment		Adolescents	5-10 min × 2	Two telephone calls or text-messages, supporting the adolescents to follow the training plan
7	Adolescents	90 min	In-session exposure and behavioral experiments. Homework assignment				
8	Adolescents	90 min	In-session exposure and behavioral experiments. Additional skills if needed to facilitate progress (e.g., problem solving, assertiveness). Homework assignment				
9	Adolescents	90 min	Troubleshooting exposure. In-session exposure. Homework assignment				
10	Adolescents	90 min	Reviewing goals. Positive and negative coping strategies. Future plans. Celebration.	5	Adolescents	45 min	Review of progress so far. Future plans Mutual support

^a^Two psychoeducational parent sessions, each 90 min. The first between session 1 and 3 and the second after session 5

## Methods

### Study design

This is a RCT with groups of 5-8 adolescents (12-16 years) allocated to two school-based CBT interventions and a control condition: (1) a brief intervention developed for the present study (VAAG), (2) a longer, more established intervention (CHILLED) and (3) a delayed access waitlist control group (WLC). Participants allocated to the two active interventions will start after a group of 5-8 adolescents has been assembled and randomized. Both interventions last for 10 weeks, VAAG comprises five sessions and CHILLED ten sessions. In addition CHILLED has two separate parent sessions. The expected number of hours of face-to-face professional contact is 18 h for the Chilled program and 5.5 h for VAAG. The waitlist group will be randomized in groups of 5-8 adolescents. Those allocated to the waitlist group will be randomized a second time to either VAAG or CHILLED after a delay of 10 weeks. Based on previous research, CBT early intervention for anxious youths is expected to be efficacious [26, 27]; therefore, we consider it unethical to withhold the intervention for adolescents allocated to the waitlist group for an extended period. Participants from all three conditions will be invited to a 1-year follow-up assessment. The overall study design is illustrated in Fig. 1, whereas the stages of the enrollment, interventions and assessments can be seen in Additional file 1: Figure S1 (The SPIRIT table).

### Participants and procedure

Participants will be students in junior high schools, between 12 and 16 years, with self- or parent-reported levels of anxiety symptoms above a set cutoff and who report that anxiety interferes with their daily life.

#### Inclusion criteria

1. An overall score of ≥25 on the anxiety inventory Spence Children’s Anxiety Scale (SCAS) [36] *and* a score of ≥1 on the first question of the Children’s Anxiety Life Interference Scale (CALIS) [37], indicating that anxiety interferes with daily life of the youth, rated by *either* the adolescents *or* one parent.
2. The adolescent and at least one parent understand and read Norwegian.
3. Assent from the youth and signed informed consent from the parent.

As part of the present study we collected data on self-reported anxiety symptoms from all adolescents in the included schools between October 2014 and June 2015. Adolescents and parents were invited to take part in a survey focusing on symptoms of anxiety and related youth mental health problems. The survey was completed in the classroom during school time, and participants received feedback about their score on the anxiety symptom measure. Adolescents with heightened levels of anxiety were informed about the present study and invited to make contact with the school nurse for further assessment for inclusion. In the survey we found a mean youth-reported SCAS score of 23.19 (*SD* = 15.60) (Raknes, Pallesen, Bjaastad, Wergeland, Hoffart, ﻿Dyregrov, Haaland, Haugland:﻿Negative life-events, social support, and self-efficacy in anxious adolescents (submitted)). In the present study the cutoff on the anxiety measure is above this mean score, allowing for the inclusion of youths with mild and moderate levels of anxiety.

#### Exclusion criteria

1. The adolescent exhibits behavior that makes participation in groups with other adolescents challenging. This is evaluated by the school nurse, based on information from the adolescent, the parent and the teacher. In each case, the school nurse makes an evaluation based on the following questions:
  - Is the adolescent able to follow group rules?
  - Will the adolescent behave in ways that disrupt the group?
  - Does the adolescent have learning problems to an extent that will make it difficult to follow the group program?

The inclusion and exclusion criteria are selected to mirror “real-life practice” as much as possible. Thus, inclusion is not based on formal diagnostic evaluation, and youths participating in other treatments (medication, other psychotherapy) are not excluded [38].

#### Recruitment

Participants will be recruited from 17 public and 1 private junior high school (8th to 10th graders between 12-16 years) located in 9 municipalities in the West, East and South of Norway. We apply several recruitment strategies to ensure a sufficient sample size. The school nurses are in charge of the recruitment as well as the baseline assessments. From a pilot study we found this to be a feasible procedure (﻿Fjermestad, Wergeland, Bjaastad, Rogde & Haugland: Indicated prevention for anxious youth: A feasibi﻿lity study in the school helath services (in preparation)). Adolescents are recruited from routine meetings with the school nurses (including all 8th graders). School nurses inform teachers about the study and how to recognize anxious adolescents in a school setting. Other primary health-services (e.g., community psychologists, school psychologists, medical doctors) receive information about the study and may nominate adolescents to be assessed for inclusion. Furthermore, information is given to parents and adolescents (e.g., by pamphlets, at school meetings, information boards and local media), making recruitment by self-referral an option. Finally participants are informed and recruited through the school survey mentioned above.

#### Enrollment and randomization

Eligible adolescents first meet with the school nurse for information about the study. This is followed by a semi-structured interview developed for this study, conducted with the adolescent and caregiver(s). The interview includes assessments of anxiety symptoms, severity and impairment, what goals the adolescent wants to achieve with regard to anxiety as well as assessment of whether exclusion criteria apply. Next, the adolescent and caregiver(s) complete a battery of questionnaires administered electronically. These may be completed either at the office of the school nurse or at home. Based on their score on the anxiety symptom measure and the interference measure, the adolescent is enrolled in the study. Randomization to condition occurs after having enrolled five to eight adolescents from the specific school, with the group serving as the unit of randomization. As 3 of the 18 schools are too small to recruit groups, youths from these three schools are included in groups at a nearby school, resulting in 15 sites for randomization. The randomization is done according to an outline prepared at the start of the study, using a computer-generated random digit procedure, comprising lists of six possibilities (2 CHILLED × 2 VAAG × 2 WLCs) at each school. After the waiting period the WLC group is randomized to CHILLED or VAAG. The randomization will be administered by staff employed at Uni Research Health. To reduce systematic biases between schools and group leaders, all three conditions will take place at each school, with group leaders being trained to deliver both CHILLED and VAAG.

#### Interventions

*VAAG* [39] is a manualized CBT program developed for this study, comprising five sessions, each session lasting between 45 and 90 min (see Table 1). The program lasts for 10 weeks and is administered as a group intervention with two group leaders. The first four meetings occur weekly and are focused on basic CBT principles for treating youth anxiety, e.g., psychoeducation, cognitive restructuring, in-session exposure exercises and behavioral experiments, as well as homework assignments (see Table 1 for further description of VAAG). Parents are invited to participate in session two together with the adolescents, where helpful parenting with anxious youths is discussed. Parents receive written material with advice on how to support their adolescent in coping with anxiety. In a period of 5 weeks, between sessions four and five, the adolescents are encouraged to practice exposure tasks on their own, assisted by self-help material and two phone calls or text messages from the group leaders. The self-help material is the Psychological First Aid kit (PF) [40], which is a central part of the VAAG program. This is a tool used for psychoeducation and communication about basic CBT principles and also used as a self-help tool between sessions. PF comprises an illustrated booklet, small figurines used as therapeutic reminders to externalize “helpful” and “non-helpful” thoughts, and a work sheet (“the helping hand”). A previous feasibility study indicates that primary health workers find PF to be a useful therapeutic tool, particularly when working with youth with anxiety and depressive symptoms [41]. VAAG ends with a fifth group meeting, reviewing each participant’s progress, setting new goals and mutual support.

*CHILLED* (adolescent version of the Cool Kids program) [42] is a manualized CBT program for the treatment of youth with anxiety [31, 32, 43]. In the present study, the school version of the program is applied. The CHILLED program addresses cognitive, physiological, emotional and behavioral components that interact in the development and maintenance of anxiety (Table 1). The program comprises separate workbooks for adolescents and parents with session summaries, worksheets and guides for home practice. CHILLED is a ten-session program for the management of broad-based childhood anxiety disorders (e.g., social anxiety, separation anxiety and generalized anxiety). Adolescents attend the sessions on a weekly basis, each session lasting 90 min. The sessions are led by two group leaders and cover standard CBT principles for the treatment of youth anxiety. In-session, therapist-led exposures are included to ensure that the exposure exercises are individualized and successful. Exposure training is also included as a homework assignment between sessions. The program includes additional skills training, not directly related to anxiety management (e.g., assertiveness training, problem solving and dealing with bullying). Parents are invited to attend two separate parent information evenings where they are informed about the content of the program and discuss ways to support their anxious child.

In the present study, the CHILLED program is administered with the same content and structure as in previous school studies, with three exceptions: we apply the adolescent version of the program, and the non-anxiety skills (e.g., assertiveness training) are voluntary for the group leaders to include. If these are not included, they will have more time for in-session exposure tasks and cognitive restructuring. We have also replaced the group meeting in session five with individual sessions (15-20-min individual session for each participant). This is done to ensure that the exposure plans are targeted to focus on the anxiety problems of each youth. These changes are made in agreement with the authors of the CHILLED program and are in accordance with their on-going revision of the program.

Time spent by school nurses in preparing, delivering and receiving supervision will be measured. If VAAG is found to be non-inferior to CHILLED with regard to effect, while less face-to face therapist time and time for preparation and supervision are needed, this will be a less resource-consuming intervention for schools to implement.

### Assessment

Adolescents and parents will complete questionnaires administered electronically at pre-treatment (T1), mid-treatment (T2), post- treatment (T3) and 12-month follow-up (T4) (see Table 2 and Additional file 1: Figure S1 (the SPIRIT table)). We plan for the group leaders to meet with the adolescents at all points of data collection (T1-T4) to secure outcome data from as many as possible, regardless of participation or discontinuation in the intervention.

**Table 2:** Overview of measures

Measure	Scale (informant)	T1	T2	T3	T4
Primary outcome measures					
Anxiety symptoms	SCAS (c/p)	●	●	●	●
Interference of anxiety symptoms	CALIS (c/p)	●	●	●	●
Secondary outcome measures					
Depression	SMFQ (c/p)	●		●	●
Quality of life	KINDL (c/p)	●		●	●
General mental health	SDQ (c/p)	●		●	●
Clinical Global Impression	CGI (g)	●		●	●
Other measures					
Age, gender, socioeconomic status, ethnicity	From the youth@hordaland-survey (c/p)	●			
Medication, service use, school absenteeism	Developed for this study (p)	●		●	●
Youth Engagement in and between sessions^*^	YES (c/g)				
Sleep problems	From the youth@hordaland-survey (c)	●		●	●
Children’s Automatic Thought Scale	CATS (c)	●	●	●	●
General Self-Efficacy Scale	GSE	●	●	●	
Family cohesion	READ subscale (c)	●			
Social support	READ subscale (c)	●			
Adverse childhood experiences	From the youth@hordaland-survey (c)	●			
Parenting psychopathology/stress	DASS (p)	●			
Group leader preference	Developed for this study (g)	●			
Caregiver strain	CGSQ (p)	●		●	●
Life events	LE (c)				●
Client Satisfaction Scale	CSS (c/p)			●	
Treatment Credibility and Expectancy Scale	(CES-c/p)**				

T1 = Baseline/pre-intervention, T2 = mid-intervention/at 4 weeks, T3 = post intervention/at 10 weeks, T4 = 1-year follow-up. c = child, p = parent, g = group leader

^*^Administered to group leaders after every session and to youths in session 3-9 (CHILLED) and session3-5 (VAAG)

^**^Administered to adolescents after session one and to parents after first parent session

*SCAS* Spence Children’s Anxiety Scale*, CALIS* Children Anxiety Life Interference Scale, *CGI* Clinical Global Impression Scale, *SMFQ* Short Mood and Feeling Questionnaire, *KINDL* Kinder Lebensqualität Fragebogen, *SDQ* Strength and Difficulties Questionnaire, *CATS* Children’s Automatic Thought Scale, *GSE* General Self-Efficacy Scale, *YES* Youth Engagement in and between Sessions, *CEQ* Credibility and Expectancy Questionnaire, *READ* Resilience Scale for Adolescents, *DASS* Depression, Anxiety and Stress Scale, *CGSQ* Caregiver Strain Questionnaire; the *youth@hordaland-survey* = a population-based survey administered by Uni Research Health, *CSS* Client Satisfaction Scale

#### Primary outcome measures

*Spence Children’s Anxiety Scale*-child and parent version (SCAS-c/p) [36, 44] is a questionnaire designed to assess youth anxiety symptoms. Both child and parent versions contain 38 items (in addition, the child version has six positive filler items). SCAS c/p consists of six 4-point subscales for specific anxiety areas: panic/agoraphobia, social phobia, generalized anxiety, separation anxiety, obsessive compulsive, and specific phobias. Each subscale can be scored separately as well as added together for an overall anxiety symptom score. SCAS c/p has been found to have good psychometric properties [36, 44–46].

*Child Anxiety Life Interference Scale*-child and parent version (CALIS-c/p) [37] assesses life interference and impairment from youth anxiety in the areas of home, social life, school and activities. In addition to a nine-item scale filled out separately by adolescents and caregivers, caregivers also rate how the child’s anxiety interferes with their own life (seven items; e.g., career, relationship with spouse). Each item is rated on a 5-point scale. CALIS has demonstrated satisfactory psychometric properties [37].

#### Secondary outcome measures

*Short Moods & Feelings Questionnaire*-child and parent version (SMFQ-c/p) [47] is a 13-item scale assessing youth depressive symptoms within the last 2 weeks. Items are rated on a 3-point scale from “not true” to “true.” The SMFQ-c/p is designed for children and adolescents aged 8-16 years. The scale has shown good psychometric properties [47–49].

The *Clinical Global Impression scale* (CGI) [50] is a measure of global functioning covering the overall severity of symptoms (CGI Severity; CGI-S) and changes in functioning over time (CGI Improvement; CGI-I). CGI-S/I is scored on a 7-point scale. In the present study, CGI-S/I will be completed by the school nurses (pre- and post-treatment). Ratings will be based on a joint semi-structured interview with caregivers and adolescents. All interviews will be videotaped, and reliability will be checked by external observers rating about 15% of the interviews.

*Strengths and Difficulties Questionnaire*-child and parent version (SDQ-c/p) [51] is a 25-item behavioral screening questionnaire assessing emotional problems, conduct problems, hyperactivity-inattention, peer problems and prosocial behaviors in children and teenagers. Items are scored on a 3-point scale. SDQ-c/p also includes a five-item scale on functional impairment scored on a 4-point scale. SDQ-c/p has demonstrated good psychometric properties [52, 53].

*Questionnaire for Measuring Health-Related Quality of Life in Children and Adolescents, The Revised Version-child and parent version *(KINDLRc/p) [54] measures quality of life. KINDL-R-c/p consists of six subscales with four items each (physical and emotional well-being, self-esteem, family, friends and school), all items scored on a 5-point scale. The German versions of KINDL-R-c/p have satisfactory psychometric properties [54], with the Norwegian translated version evaluated as promising [55, 56].

#### Other measures

Further measures are included to allow for descriptive information about the participants as well as analyses of moderators and mediators. *Socio-demographic information* comprises gender, age, ethnicity, parents’ education and family economy, as well as the use of health services, medication and school absenteeism. *Sleep problems* covers sleep duration, difficulties initiating and maintaining sleep, and tiredness/sleepiness, all derived from the youth@hordaland-survey [57, 58]. The *General Self-Efficacy Scale* (GSE) [59] assesses optimistic self-beliefs, whereas the *Children’s Automatic Thoughts Scale* (CATS) [60] assesses negative thoughts associated with mental health problems in youths. The *Treatment Credibility and Expectancy Scale* (CES-c/p) [61] refers to how believable, convincing and logical a treatment is perceived to be, while treatment expectancies refer to the improvements believed to be achieved. The *Client Satisfaction Scale* (CSS-c/p) assesses both parents’ and adolescents’ evaluation of the program. *Youth Engagement in and between Sessions* (YES) assesses the adolescents’ participation in in-session exercises and discussions and exposure training between sessions. *Adverse life experiences* cover the occurrence of serious accidents such as violence or unwanted sexual acts and bullying, previously applied in the youth@hordaland-survey [62]. A *Life-event Scale* (LE) comprises positive (e.g., sporting achievement), negative (e.g., family member becomes ill) and neutral events (e.g., change school) and is completed by the adolescents at 1-year follow-up. The *Resilience Scale* (READ) [63] measures the ability to handle stress and negative experiences. In the current study the subscales “Social resources” and “Family Cohesion” are included. The *Depression Anxiety Stress Scale* (DASS-21) [64] is a self-reported measure assessing parental symptoms of anxiety, stress and depression. The *Caregiver Strain Questionnaire* (CGSQ) [65] measures the negative impact on parents of caring for a child with emotional or behavioral problems. Finally, after completing the initial training group, leaders rate which of the two programs they prefer.

### Program implementation

#### Training and supervision of group leaders

Two group leaders will run each group, with one group leader and one co-leader. The main group leaders (*n* = 18) will be school nurses (with the exception of two mental health workers from primary health services). The co-leaders will be school nurses (*n* = 11), community psychologists (*n* = 3) and mental health workers from community mental health outpatient clinics (*n* = 5). At the start of the study the group leaders receive a 4-day skills-training workshop comprising basic CBT principles for youth anxiety, introduction to VAAG and CHILLED and training in how to conduct the pre-post assessment interviews. Additional training is given regularly during the inclusion period, with two 2-day workshops (i.e., on youth anxiety, recruiting participants, exposure training, cognitive restructuring and group processes).

#### Strategies to improve and measure adherence and competence

Supervision will be provided throughout the study. All sessions will be videotaped, and group leaders will receive feedback based on videotapes of sessions and according to a detailed supervision plan. The supervision plan entails information on the duration, structure, number and content of the supervision sessions. Supervision varies between 3 and 4.5 h for VAAG and 6 and 10.5 h for CHILLED per group depending on the number of groups the group leaders have administered. The supervisors are experienced CBT therapists, with a background in clinical child psychology, community psychology or child psychiatry. Decisions about discontinuing or modifying the interventions (e.g., referral to specialist treatment) will be discussed with the supervisor in each individual case. Videotaping the sessions allows for ratings of adherence and competence in the delivery of the programs. Between 20 and 40% of the videotaped sessions will be rated according to a scale for rating adherence and competence [66]. The scale has adequate psychometric properties and has previously been used in a CBT treatment study for anxious youth [67]. The scale assesses CBT structure, process and relational skills as well as specific goals for each session. School nurses volunteer for the study and do not receive extra pay, but receive free training and supervision. Some of the school nurses will be relieved from other duties to participate in the current study (about 20% less workload).

### Statistical analyses

#### Power calculation

Power calculations for differences among the three conditions, taking into account two repeated measurements with an assumed correlation of 0.6, yielded a required total sample size (power = 0.80, alpha = 0.05) of 294 children to obtain an effect size (ES) of 0.40. With an assumed attrition of 10%, we need to recruit 323 participants. Even though an average effect size in targeted prevention studies is 0.32 [26], we expect a somewhat larger effect size compared to WLC since we have included an intervention that has shown moderate to large effect sizes in previous studies when delivered as treatment or as a targeted preventive school intervention [31–33].

#### Non-inferiority

The study has two primary outcome measures, each with caregiver and youth self-report versions (SCAS-c/p and CALIS-c/p). For each of the two scales a non-inferiority limit was determined based on an assumed difference in effect size of 0.1 between the interventions. This is the average ES found between two active preventive interventions with anxious youth [26]. Based on an effectiveness study of CBT for anxious youth in Norway [67], a SD of 14 will be applied for the SCAS-c/p post-intervention scores. With a difference in ES = 0.1, a non-inferiority bound is set to 1.4. This means that if the difference in post score in SCAS between VAAG and CHILLED is ≤1.4 in a one-sided test with α = 0.025, then VAAG will be found non-inferior to CHILLED on the SCAS scale. For CALIS-c/p (anxiety interference on child’s life), the non-inferiority bound is set to 0.7, based on results from an RCT efficacy study with anxious youth in Denmark [31] where an SD = 7 was found on CALIS c/p. Furthermore, to conclude that VAAG is non-inferior to CHILLED, three out of four primary outcome measures (SCAS-c/p and CALIS-c/p) have to be declared non-inferior.

#### Data analytical plan

Data will be collected electronically. The effects of the two active interventions compared to the WLC will be analyzed by mixed effect models. The predictive value of the various pre-treatment characteristics will be evaluated using regression analyses. The test for non-inferiority will be performed for the primary outcome variables, followed by superiority analyses, including both primary and secondary outcome measures. Analysis will be performed according to an intention-to-treat procedure. Missing data will be handled by multiple imputations. Further details about the protocol are reported in the SPIRIT checklist (Additional file 2: Table S1).

## Discussion

Prevention and early intervention approaches to anxiety disorders are important, as young people often experience barriers toward seeking mental health services. The present study will provide information about the efficacy of school-based CBT interventions for anxious youth. If they are found to be effective, these interventions could be subject to large-scale implementation in school health services. The study extends previous studies on early intervention for anxious youth by comparing two programs with different intensity. The brief intervention may be easier to implement in schools as it requires less time and professional resources, but needs first to be found non-inferior to the more intense intervention. In the present study, psychometrically sound measures are applied with multiple informants in a randomized controlled design. The interventions are studied with conditions that closely match real-world implementation. The use of diagnostic interviews and blind evaluators of treatment gains was, however, not feasible with the resources available and would also not be in accordance with the school health services as they are usually being provided.

### Trial status

Recruiting. Inclusion of participants started in October 2014. By November 2016, 310 adolescents have been included and randomized into the study. Inclusion will be finished by December 2016 and data collection by March 2018.

## Additional files

Additional file 1: Figure S1. The SPIRIT table. (DOC 51 kb)
Additional file 2: Table S1. The SPIRITchecklist. (DOC 105 kb)

## Abbreviations

CALIS: Children Anxiety Life Interference Scale
CATS: Children’s Automatic Thought Scale
CBT: Cognitive Behavioral Therapy
CEQ: Credibility and Expectancy Questionnaire
CGI: Clinical Global Impression Scale
CGSQ: Caregiver Strain Questionnaire
DASS: Depression, Anxiety and Stress Scale
ES: Effect Size
GSE: General Self-Efficacy Scale
KINDL: Kinder Lebensqualität Fragebogen
LE: Life-Event scale
LI-CBT: Low-Intensity CBT
PF: Psychological First Aid kit
RCT: Randomized Controlled Trial
READ: Resilience Scale for Adolescents
SCAS: Spence Children’s Anxiety Scale
SDQ: Strength and Difficulties Questionnaire
SMFQ: Short Mood and Feeling Questionnaire
WLC: Waitlist Control Group
YES: Youth Engagement in and between Sessions

## Footnotes

1 VAAG is Norwegian for both to dare and to venture.
