# Supplementary material for: School-based cognitive behavioral interventions for anxious youth: study protocol for a randomized controlled trial
Source: Trials. 2017 Mar 4;18:100. doi: 10.1186/s13063-017-1831-9 (PMC5336667; doi:10.1186/s13063-017-1831-9)
Supplement: Additional file 1: Figure S1. — The SPIRIT table. (DOC 51 kb) [file 13063_2017_1831_MOESM1_ESM.doc]

Additional file 1: Figure S1. SPIRIT table for the evaluation of two school based CBT interventions

|  | **STUDY PERIOD** | | | | | | |
| --- | --- | --- | --- | --- | --- | --- | --- |
|  | **Enrolment** | **Allocation** | **Post-allocation** | | | | **One year follow up** |
| **TIMEPOINTS** | ***T1***  ***pre*** | **0** | ***Weeks***  ***1-4*** | ***T2***  ***mid*** | ***Weeks 5-10*** | ***T3***  ***post*** | ***T4***  ***FU*** |
| **ENROLMENT:** |  |  |  |  |  |  |  |
| **Eligibility screen^a^** | X |  |  |  |  |  |  |
| **Informed consent** | X |  |  |  |  |  |  |
| ***Baseline assessment*** | X |  |  |  |  |  |  |
| **Allocation^b^** |  | X |  |  |  |  |  |
| **INTERVENTIONS:** |  |  |  |  |  |  |  |
| ***Intervention A: CHILLED*** |  |  |  |  |  |  |  |
| ***Intervention B:***  ***VAAG*** |  |  |  |  |  |  |  |
| ***Wait-List control group*** |  |  |  |  |  |  |  |
| **ASSESSMENTS:** |  |  |  |  |  |  |  |
| ***Primary outcomes (SCAS, CALIS)*** | X |  |  | X |  | X | X |
| ***Secondary outcomes***  ***(SMFQ,CGI,***  ***KINDL, SDQ)*** | X |  |  |  |  | X | X |
| ***Mediators and/or moderators***  ***(e.g CATS,***  ***GSE, CGSQ)*** | X |  |  | X |  | X | X |

^a^ Study eligibility for the individual adolescent is based on scores on the SCAS c/p and the CALIS c/p questionnaires assessed at T1 (primary outcome measures)

^b^ Allocation (randomization) occurs when 5-8 adolescents have been assessed and found eligible according to inclusion criteria
